# Diet-Microbiota Interactions and Their Implications for Healthy Living

**DOI:** 10.3390/nu5010234

**Published:** 2013-01-17

**Authors:** Ian B. Jeffery, Paul W. O’Toole

**Affiliations:** 1 Department of Microbiology, University College Cork, College Road, Cork, Ireland; E-Mail: pwotoole@ucc.ie; 2 Alimentary Pharmabiotic Centre, University College Cork, College Road, Cork, Ireland

**Keywords:** microbial, diversity, IBS, ageing, diet, microbiota, microbiome, SCFA, vitamins

## Abstract

It is well established that diet influences the health of an individual and that a diet rich in plant-based foods has many advantages in relation to the health and well-being of an individual. What has been unclear until recently is the large contribution of the gut microbiota to this effect. As well as providing basic nutritional requirements, the long-term diet of an animal modifies its gut microbiota. In adults, diets that have a high proportion of fruit and vegetables and a low consumption of meat are associated with a highly diverse microbiota and are defined by a greater abundance of *Prevotella* compared to *Bacteroides*, while the reverse is associated with a diet that contains a low proportion of plant-based foods. Furthermore, it is becoming increasingly clear that the effect of the microbial ecology of the gut goes beyond the local gut immune system and is implicated in immune-related disorders, such as IBS, diabetes and inflamm-ageing. In this review, we investigate the evidence that a balanced diet leads to a balanced, diverse microbiota with significant consequences for healthy ageing by focusing on conditions of interest.

## 1. Introduction

The gastrointestinal tract contains a diverse microbial community, which is predominantly bacterial and which is referred to as the gut microbiota. Over the last decade, the microbial composition of the gut has been the subject of increasingly intense research, much due to its demonstrated impact upon various health conditions. The Human Microbiome Project (HMP), MetaHIT (Metagenome of Human Intestinal Tract) and the smaller ELDERMET project, as well as numerous labs around the world (Table 1), have taken advantage of new high-throughput technologies to thoroughly characterise the human microbiome from multiple body sites at every stage of life. A number of these projects employed whole-community shotgun sequencing to study the gene content of the microbial populations. Through these studies, it has become apparent that these microorganisms are not just passive residents of the gut, but carry out a range of biological functions that are important in the nutrition and well-being of the individual.

**Table 1:** Microbiome consortiums.

Program	Duration	Funding Organization	Conditions of Interest
*NIH Jumpstart Program*	2007–2008	NIH, USA	Generate 200 complete bacterial genome sequences and perform compositional analysis of various body regions.
*NIH Human Microbiome Project*	2007	NIH Roadmap Program, USA	Characterize the microbes on the human body and correlate the changes in these microbial populations with human health.
*DACC*—*Data Analysis and Coordination Center*	2008–2013	NIH Human Microbiome Project (HMP), USA	Assist in standardization of data pipelines (storage, analysis and display of data) and provide access to data.
*MetaHIT*, *Metagenomics of the Human Intestinal Tract*	2008–2011	European Commission (FP7)	Describe the role of the microbiota in Inflammatory Bowel Disease (IBD) and obesity, and generate a reference catalogue of intestinal microbial genes.
*Canadian Human Microbiome Initiative*	2009	Canadian Institutes of Health Research (CIHR), Canada	A number of projects relating to human microbial interactions and their effect on health.
*The Australian Jumpstart Human Microbiome Project*	2009	Commonwealth Scientific and Industrial Research Organisation (CSIRO), Australia	Sequencing of specific bacterial strains and the application of metagenomics techniques to investigate the interaction between intestinal microbes and their host.
*MicroObes*, *Human Intestinal Microbiome in Obesity and Nutritional Transition*	2008–2010	French National Agency for Research (ANR), France	Identify metagenomic signatures that characterise the relationship between the intestinal microbiota and the nutritional and metabolic status of the host.
*Korean Microbiome Diversity Using Korean Twin Cohort Project*	2010–2015	National Research Foundation of Korea, Korea	Determine the microbiomes on various epithelial sites of the human body using Korean Twin Cohort and investigate the relationship between human microbiomes and disease. To establish a dedicated centre for Korean microbiome information and analysis.
*ELDERMET Project*	2007–2013	National Development Food Research Health Initiative and Science Foundation Ireland	Characterize the faecal microbiota associated with ageing and correlate diversity, composition, and metabolic potential of the faecal microbial metagenome with health, diet and lifestyle.

The most heavily studied part of the gut is the distal colon, through the use of high-throughput next-generation technologies and phylochips to characterise faecal derived populations. In adults, this population is characterised by the predominance of two phyla, *Bacteroidetes* and *Firmicutes*, with minor contributions from the *Proteobacteria* and *Actinobacteria* [1]. However, it is known that the microbial content of the gut changes along its length, with the small intestine containing a higher relative abundance of members of the *Actinobacteria* and *Streptococcaceae* [2]. Autochthonous bacteria have evolved within the gut to form a mutually beneficial relationship with their human hosts. Considering the size of the human gut and the density of bacteria that it contains, the nature of this relationship is very relevant. The bacteria in the gut have developed a number of adaptations for living in this unique environment, such as mechanisms for avoiding or modulating inflammatory responses [3]. On the other side of this interaction paradigm, evolution in the host has taken the form of Toll-like receptors (TLR) that are used to identify bacteria (and other antigens) and to respond appropriately as part of an innate and adaptive immune system [4]. The host also produces a complex polymeric barrier or mucin layer that separates the bacteria from the epithelium [5,6]. A number of studies have examined the core human microbiota within the gut, which may be operationally defined as those microbes that are found in a majority of individuals [7]. Qin *et al*. (2010) [8] found that there are 1000 to 1150 prevalent bacterial species, with each individual harbouring at least 160 species. This translated to 3.3 million non-host-encoded genes of which 99% were of bacterial origin, with ~536,000 prevalent unique bacterial genes in each individual of which 40% would be shared with at least half of the individuals in their cohort. Annotation suggests that these microbial genes are involved in the production of essential metabolites and the degradation and utilisation of complex carbohydrates and biosynthesis of vitamins [8,9].

There are significant inter-individual differences in the presence and absence of the bacterial species found in the gastrointestinal tract. There are a number of highly abundant species that are present in the majority of a population, but the overall composition of the gut depends on the age, health, diet and even geographical location of the individual [10]. These bacteria have a substantial scope to modify the phenotype of the individual though production of metabolites and through interaction with the physiology of the individual in a diverse number of ways. Thus, dietary modulation of the gut microbiota composition and function will have implications for the host phenotype.

## 2. Microbiota and Diet

A number of studies have examined the relationship between the faecal microbiota and diet. De Filippo *et al*. (2011) [11] showed that the faecal microbiota in a cohort of Italian children was different to that found in children in a rural village in Burkina Faso. Similar to the different microbial populations reported by the ELDERMET consortium [12], Italian children, who consume less plant-based foods, harboured more *Bacteroides* and *Alistipes* in their microbiota. The microbiota of the rural Burkina Faso children had an abundance of bacteria from the genus *Prevotella*, similar to the healthier diet individuals in the ELDERMET cohort. The Burkina Faso children also harboured significantly higher levels of faecal short-chain fatty acid producers than the Italian children. Interestingly *Xylanibacter*, which was absent in the ELDERMET and Italian microbiota communities, but was present in the Burkina Faso children, is known to contain a set of genes for cellulose and xylan hydrolysis. The main fibres that reach the distal colon and are utilized by the gut microbiota and their enzymes are carbohydrates and proteins that are refractory to host digestion. The level of metabolic activity in the microbiota will depend on how much of these materials are making it down into the gut. Although the microbiota will respond to short-term changes in the diet, the long-term changes seem to define what type of microbial population is present and this in turn determines the metabolites produced and the potential impact on the health of the host [13]. 

Filippo *et al*. (2011) [11] hypothesized that the gut microbiota co-evolved with the polysaccharide-rich diet of Burkina Faso individuals, allowing them to maximize energy intake from dietary fibre, while also dampening inflammation and protecting them from non-infectious colonic diseases. Wu *et al*. (2011) [13] also found that the microbiota composition was strongly associated with long-term diet, with *Bacteroides* being associated with diets enriched in animal products. The *Prevotella* genus was associated with diets that contained more plant-based foods. All three studies (older persons—ELDERMET [12]; Burkina Faso versus Italian children [11]; and American adults [13]) found similar microbiota-diet associations at three different stages of life, showing that the diet modulates the microbiota regardless of gender or other confounding factors. 

A study performed in the mouse model also showed that switching from a low-fat, plant polysaccharide diet to a high fat, high sugar, Western-style diet lead to alterations in the microbiome and its metabolic function [14]. The high plant polysaccharide diets are full of resistant starch and oligosaccharides that the microbiota can utilise [15] and have been shown to affect the abundances of a number of microbes [16]. Subjects fed resistant starch type 2 from high amylose maize showed an increased abundance of *Ruminococcus* spp. and *Eubacterium rectale*, while consumption of resistant starch type 3 (water-insoluble semi-crystalline structures formed from amylose) promoted the growth of *Eubacterium rectale*, *Roseburia* spp. and *Ruminococcus bromii*. These species are able to bind directly to insoluble starch particles and to degrade them and may be the key species in bacterial food chains that target starch [17]. A detailed description on the mechanisms involved in the breakdown of fibre is beyond the scope of this review, but has been recently described [15].

## 3. Age-Related Gut Microbiota Changes

The microbiota composition is not stable over the whole life-time of an individual. At the extremities of life, a number of discernible patterns of microbiota change have been recorded. Since the microbiota and its metabolic activities react and change according to diet, some of these age-related microbiota composition changes may be attributable to diet. The diet in elderly individuals can change for a number of reasons, including loss of sensation of taste and smell, tooth loss and chewing difficulties. These changes may result in an increased consumption of high sugar/high fat foods and a reduction in the proportion of plant-based foods consumed. Although diet-related microbiota changes undoubtedly occur at all life stages, the majority of the research performed to date has been focused on healthy or diseased adults, with fewer investigations having been carried out on children and the elderly. However, in the younger and older subjects in the population, the observed microbial changes can be more dramatic, and so these are an excellent source of information on associations between health and the microbiota. This is particularly true for the study of the development and decline of microbial populations. How these populations change depends on how they are modulated. A recent study by the ELDERMET consortium has focused on elderly individuals, whose dietary patterns were found to change for a number of reasons, one of the most dramatic being the change in diet going into long-term residential care [12]. Within and between the community living subjects and those in long-term care, we uncovered an association between an increase in the microbiota diversity in the gut and a diet rich in “healthy foods”, as defined by the Healthy Food Diversity index (HFD [18]). Conversely, diets with a low HFD score were associated with microbiota populations that were less diverse and had predicted reduced functionality and so potentially conferring fewer benefits to the respective individual. As well as a difference in diversity levels, there were differences in the dominant genera present in the different subject groups. The microbiota associated with diets rich in plant-based foods were abundant in *Prevotella*, while the individuals with a lower HFD harboured more *Bacteroides*. A further reduction in the HFD scores associated with individuals living in long-term care resulted in *Parabacteroides *and *Alistipes* becoming the dominant *Bacteroidetes* genera. These microbiota alterations were associated with changes in frailty, inflammation and altered abundances of short chain fatty acids (SCFAs) producers, as well as correlations with other clinical markers [12].

Intestinal microbiota composition may also vary in elderly subjects independently of diet for a number of physiological and immunological factors, such as a reduction in the immune system functionality [19,20,21]. This is indicated in the reduced abundance of *Ruminococcus* and *Blautia* spp. and the increased abundance of *Escherichia* in the elderly when compared to young controls [12]. It has been previously reported that the microbiota associated with elderly individuals has a reduced abundance of several butyrate producers (Key members of *Clostridium *cluster XIVa and *Clostridium *cluster IV) when compared to younger individuals [22,23]. This was confirmed in the ELDERMET study, where it was shown that the oldest and frailest individuals had significantly lower copy numbers of genes related to butyrate, acetate and propionate production in the faecal metagenome [12]. The age-related increase in the proportion of facultative anaerobes, including *Escherichia* amongst other genera, has also been noted in previous studies. These bacteria are present at low levels in healthy young adults, but are present in increased abundances in the inflamed gut [22,24,25].

## 4. Dietary Modulation

As global life expectancy increases, it is important to maintain quality of life and independence in the elderly. The study of older persons is starting to provide a wealth of information and more complete understanding of their diet-microbiota interactions. This provides the possibility of modulating the microbiota to benefit health. The mechanisms of dietary modulation of the microbiota include the physical ability of high fibre diets to increase faecal bulk, which is one of the factors that affects transit time along the gut. Jumpertz *et al*. (2011) [26] have shown that the human gut microbiota can undergo long-term changes that occur over a couple of days when there is a significant increase in the calorie intake of the individual. Transit time itself affects the composition of the human faecal microbiota because, as the transit time decreases, there is a decrease in the proportions of slow growing species, such as methanogens, and an increase in sulphate reducing bacteria counts, while concentrations of total SCFAs are increased, leading to a lowering of faecal pH [27]. But, more importantly, the macronutrient composition of the ingested food determines which species flourish and which taxa will be able to colonise the gut and, so, determines the eventual composition of the microbiota.

For new taxa to become established and abundant in a stable gut ecosystem, their functionality must permit them to thrive in the presence of other organisms that are competing for resources. At the most basic level, this functionality is conferred by the presence and absence of genes. Autochthonous bacteria have coevolved with the human host and each other to perform important roles in nutrient and energy extraction and energy regulation that the host would otherwise be unable to accomplish. There are numerous positive interspecies associations in the gut [12,28], probably due to adaptation to similar substrate-availability and flow-rate environments and to cross-feeding between different organisms [29]. The importance of cross-feeding between different organisms was demonstrated by Samuel and Gordon [30] when they investigated the utilization of polysaccharides by *Bacteroides thetaiotaomicron *in germ-free mice colonized in the presence and absence of *Methanobrevibacter smithii *or the sulphate-reducing bacterium *Desulfovibrio piger*. They found that *M. smithii* directs *B. thetaiotaomicron* to focus on fermentation of dietary fructans to acetate, whereas *B. thetaiotaomicron*-derived formate is used by *M. smithii* for methanogenesis. *B. thetaiotaomicron*–*M. smithii* co-colonization resulted in increased weight gain and energy harvest when compared to mice colonised by *B. thetaiotaomicron* alone or by *B. thetaiotaomicron* and *D. piger*. Indeed, this type of crossfeeding is an important and widespread phenomenon [12,28,31]. This suggests that targeted dietary modulation of species will affect populations of microbes within the microbiota and, so, modulate energy harvest, storage and expenditure. The microbiota also affects energy balance through metabolites that its members produce. The produced metabolic outputs from the community will depend on what species/genes are present and expressed and what substrates are available to the microbiota [32].

## 5. Production of Metabolites

The gut microbiota performs many functions in the breakdown of complex carbohydrates and the generation of beneficial metabolites. This is made possible by the large array of genes that encode enzymes that are essential to these processes. Germ-free animals that do not contain a microbiota/microbiome still survive and reproduce, but their diet must contain a higher quantity and diversity of essential nutrients to maintain their health [33]. 

In conventional mice and normal humans, resistant starch and oligosaccharides that are undigested in the small intestine may be utilised in the large intestine by bacteria to produce SCFAs. There are a number of SCFAs that are produced; the most abundant being acetate, propionate and butyrate [34]. These are taken up by the host and may provide up to 10% of its energy requirements. Butyrate is absorbed by colonocytes and other cells of the colonic epithelium [35], thus providing energy to the host from dietary components that would otherwise pass undigested through the gut. In fact, if colonocytes are deprived of butyrate, they undergo autophagy [36]. Acetate and propionate are taken up by the circulatory system and, so, are utilized by the general physiology, *i.e.*, not just the intestine. Acetate is oxidized in the citric acid cycle [37], and propionate is incorporated into glucose metabolism. As well as being absorbed by the host, SCFAs have been shown to affect transit time through modulation of gut motility [38,39] and to affect insulin sensitivity and energy expenditure, giving them a link to metabolic syndrome [40].

High amounts of SCFAs may decrease the intestinal pH [41] and prevent the growth of potentially pathogenic bacteria, such as *E. coli* and other members of the *Enterobacteriaceae* [42]. While a number of gut bacteria are tolerant of lower pH values, such as a number of the *Firmicutes* species, especially those belonging to *Clostridium *cluster XIVa [43], this low pH tolerance is not a feature of many *Bacteroidetes *spp. and *Bifidobacterium *spp. [43], thus making SCFA production a potential modulator of these taxa. The increased concentration of SCFAs may also help in the absorption of minerals, such as calcium, by increasing their solubility and increasing the expression of calcium binding proteins [44]. Butyrate also exerts an anti-inflammatory [45] and anti-carcinogenic effect [46] in these cells. SCFAs may also be involved in the control of appetite, as they can be detected by receptors expressed in the gut called free fatty acid receptor 2 (FFAR2) and free fatty acid receptor 3 (FFAR3) [47]. These, in turn, regulate hormones involved in appetite control; thus, the microbiota and its production of SCFAs may have a role to play in food intake of an individual [47].

The microbiota is involved in the production and absorption of essential vitamins and micronutrients. It has been established that the levels of essential vitamins, such as folate and biotin, may be higher in individuals than their diet would suggest/support. This can be attributed to microbial production of folate and biotin, as well as a number of vitamins, including other water soluble B vitamins and vitamin K [48]. It was assumed that, as these vitamins were produced in the colon, they would not be absorbed, but excreted. This notion was challenged with the discovery of colonic transporters for biotin [49], thiamine [50], riboflavin [51], pyridoxine [52] and folate [53].

Folate is important in synthesis, repair and methylation of DNA and is necessary for cell division and growth. The primary mechanism for dietary folate absorption is its deconjugation to monoglutamates and subsequent absorption by active transport carriers in the small intestine, but evidence suggests that a similar mucosal transport mechanism operates in the colon [53]. Many microorganisms in the large intestine are capable of synthesizing folate. Furthermore, it has been shown that the infant colon contains pools of readily absorbed monoglutamylated folate [54]. A number of studies have indicated that resistant carbohydrate is utilized by the bacteria in the colon in the biosynthesis of folate, particularly *Bifidobacterium bifidum* and *Bifidobacterium longum* subsp. *infantis* [55,56,57]. A number of other B-group vitamins that are synthesised by members of the microbiota and that may be produced in the gut include riboflavin, vitamin B12, niacin and pyridoxine [58].

Vitamin K is a group of structurally similar, fat-soluble compounds that are important co-factors in the post-translational modification of certain proteins required for blood coagulation and in metabolic pathways. Microbial conversion of the plant derived vitamin K1 (phylloquinone) to vitamin K2 (menaquinone) is believed to be facilitated by a number of species in the human gut [59,60,61].

## 6. Diet, Microbiota and Functional Bowel Disorders

There is growing interest in the concept of dietary modulation of the microbiota for improving health. In modern society, disorders with plausible links to microbiota alterations are increasing, such as Irritable Bowel Syndrome (IBS) and obesity [14,62,63,64]. Irritable Bowel Syndrome (IBS) is the most common of the functional bowel disorders and affects a significant percentage of the population of the industrial world [65]. Although originally thought to be a psychosomatic disorder, it is now regarded as multi-factorial, with genetic, neurological and psychosocial elements, all potentially contributing to symptomology [66]. It is becoming increasingly recognised that low-grade inflammation [67,68] and changes in the gut microbiota are present in a majority of patients, reinforced by observations that probiotic and antibiotic intervention may be effective in reducing the severity of the IBS symptoms [69,70]. A microbiota re-configuration associated with the majority of IBS-related microbiotas has been confirmed by several studies using high throughput technologies to determine the alterations of the microbiota of the distal bowel in IBS compared to healthy subjects [63,71,72,73].

Dietary modulation of the microbiota in IBS and functional bloating is of particular interest due to the reported decrease in symptoms associated with the use of the Low FODMAP diet in the treatment of IBS [74]. FODMAP is an acronym derived from “Fermentable, Oligo-, Di-, Mono-saccharides and Polyols” [75]. This diet focuses on reducing the consumption of rapidly fermentable, short-chain carbohydrates that are poorly absorbed in the small intestine, namely fructose (monosaccharide), lactose (disaccharide), fructans and galactans (oligosaccharides) and polyols, such as sorbitol, maltitol, xylitol and mannitol [76]. Poor absorption occurs for a number of reasons; (a) fructose is only slowly absorbed due to low-capacity transport mechanisms across the epithelium [77]; (b) there is a lack of hydrolases of fructans and galactans; and (c) there are no specific transporters for polyols and these molecules are too large for uptake by passive diffusion. For lactose, a proportion of the population is unable to hydrolase the molecule, but this proportion depends on ethnicity [78].

The FODMAPs diet removes these rapidly fermentable substrates altogether or uses breath tests to stratify individuals based on their absorption of fructose and lactose [79]. Interestingly, this test has been also used to detect small intestinal bacterial overgrowth (SIBO; [80]), the eradication of which has been shown to alleviate the symptoms of IBS [65]. There is limited data to suggest that fructose malabsorption and SIBO might have a bidirectional cause-effect relationship, but nevertheless, upon the administration of fructose, there is an increase in the hydrogen produced as measured by the breath test and an increase in the concentration of acetate found in the blood [76], which may be produced by *E. coli* [81]. It has been shown that a diet that contains no substrates for bacteria to ferment alleviates IBS symptoms in 85% of cases, accompanied by a normalisation of the lactulose breath test, an indicator of SIBO [82].

At the time of writing, there is no information on how consumption of a FODMAPs diet would affect the microbiota signature detected in faecal samples of IBS subjects. It has been shown that when individuals are fed a high FODMAP diet, a significant proportion of the substrates will pass through the small intestine to the proximal colon [83]. This increased substrate in the proximal colon may explain the reported increased microbial production of SCFAs by the microbiota of IBS subjects [84,85,86]. Many of the faecal microbiota taxa that have been shown to be associated with IBS, such as *Ruminococcaceae* and *Clostridium *cluster XIVa, are known to be enriched for species that produce SCFAs [87]. As described previously, SCFAs are essential to the normal functioning of the gut, but there is some evidence that abnormally high levels of butyrate can promote visceral hypersensitivity [88] and high levels of SCFAs can cause powerful contractions in the terminal ileum [89]. These observations are of clinical relevance, given the association of IBS with visceral hypersensitivity and dysmotility. 

The FODMAPs diet alleviates the symptoms of IBS better than other healthy eating advice, with the majority of individuals on the diet reporting improved symptoms [90]. It has not been shown that the diet normalises the individual, and we may speculate that a resumption of the normal diet will result in a resumption of the original symptoms. However, the FODMAPs diet has been shown to be an effective way to alleviate symptoms of functional gastrointestinal disorders. 

With regard to food related issues that may have a causative effect, there is a perception among IBS sufferers that they may suffer from food intolerance or allergy, and this leads many IBS subjects to initiate a variety of dietary changes [91]. Altering of diets in this regard is common, with 20%–70% of IBS sufferers self-reporting one or more food intolerances [92]. Despite this, clinically validated food hypersensitivity appears to be relatively uncommon in IBS [93], but, when detected, the removal of the offending foods has been associated with a reduction or a complete elimination of symptoms [94,95]. However, the mechanism of action of the FODMAPs diet may explain why IBS symptoms may be eased by a gluten free diet [96], because this diet is low in content of cereals that contain high levels of FODMAP substrates.

## 7. Obesity and Metabolic Disorder

Although the development of obesity and insulin resistance is complex, with many genetic and environmental factors involved [97,98,99,100,101,102,103], it is associated with the presence of chronic inflammation in visceral adipose tissue, which is believed to be a leading promoter of insulin resistance in obesity. Studies, primarily in animals, have demonstrated that a number of microbial factors may be associated with the development of metabolic disorder and diabetes [104]. Cani *et al*. (2008) [105] showed that genetically obese ob/ob mice, which are characterised by obesity and insulin resistance, showed an improvement in insulin sensitivity and inflammatory parameters when treated with antibiotics. 

Turnbaugh *et al*. (2009) [106] reported that obesity is associated with microbial compositional changes at the phylum level. They found that individuals with high BMIs had a lower proportion of *Bacteroidetes* and a higher proportion of *Actinobacteria* when compared to leaner individuals. This agreed with their previous study on obese mice, where they found a 50% reduction in the abundance of *Bacteroidetes* and a proportional increase in *Firmicutes* [107] and also agreed with major findings of another study of humans in 2006, where they detected a decreased relative proportion of *Bacteroidetes* in obese individuals [108]. However, a number of studies have failed to confirm these associations and have reported the opposite association [109] or found no association at all [26,110]. 

Studies have shown that the composition of the diet and the amount of calories consumed are strong modifiers of the microbiota. Zhang *et al.* (2010) [111] examined genetic susceptibility, diet and weight gain and how they affect the microbiota in Apoa-1 knockout mice compared to wild-type and reported that the majority of the microbiota changes that were detected were associated with the diet [111]. This dietary effect was almost five-times the effect of the genetic susceptibility, reinforcing the traditional view of the dietary impact on weight. However, without a microbiota, germ-free animals are resistant to high-fat diet-induced obesity and metabolic syndrome [112] and genetically identical animals respond differently to a high fat diet [113]. This phenotypic independent association of the microbiota was demonstrated using Sprague-Dawley rats that were found to be obesity-prone or obesity-resistant, depending on their microbiota composition. The obesity-prone rats exhibited increased TLR4 activation, which was associated with ileal inflammation [114], as well as a decrease in intestinal alkaline phosphatase, a luminal enzyme that detoxifies lipopolysaccharide (LPS). This resulted in increased localization of occludin in the cytoplasm of epithelial cells and phosphorylation of the myosin light chain, which is associated with increased intestinal permeability [114]. Furthermore, there were increased concentrations of plasma LPS in the circulatory systems of the rats. It was concluded that the consumption of a high-fat diet induces the changes in the gut microbiota, but the development of inflammation was associated with the appearance of hyperphagia and an obese phenotype. This was confirmed in a subsequent study, where it was shown that infusing a low level of LPS for four weeks caused weight gain and insulin resistance without a high-fat diet [115]. Further evidence was provided by Serino *et al.* (2012) [116], who showed that when genetically identical mice were fed a fat-enriched carbohydrate-free diet, not all mice developed insulin resistance. The subgroup of mice that did show a marked change in insulin sensitivity also presented with a modified gut microbiota. This microbiota (and the increased insulin resistance) was associated with increased gut permeability, increased endotoxaemia and systemic/adipose tissue inflammation. The associated global microbiota changes can be summarised as an inverted *Bacteroidetes* to *Firmicutes* ratio with a reduction in the proportional abundance of the *Lachnospiracaea* family and *Oscillibacter* genus in the insulin resistant subgroup. Qin *et al.* (2012) [117] described a decrease in the abundance of butyrate-producing bacteria and an increase in various opportunistic pathogens, as well as an enrichment of other microbial functions conferring sulphate reduction and oxidative stress resistance in diabetic individuals. They did not, however, comment on the association with diet.

The microbiota also affects the expenditure and storage of the nutrients through the expression of genes in the host, such as fasting-induced adipocyte factor (Fiaf). Fiaf is a circulating lipoprotein lipase inhibitor that induces circulating lipoprotein lipase to hydrolyse circulating triacylglycerols to free fatty acids [118]. Bäckhed *et al*. (2007) [112] investigated obesity associated with a western-style, high fat, high sugar diet in mice and showed that mice responded with increased adiposity, but that animals that were germ-free were protected from obesity. This resistance disappeared in germ-free knockout mice lacking the Fiaf gene and, so, were not protected from the diet-induced obesity. This evidence points to an unfavourable dietary modification of the microbiota being related to the onset of hyperphagia, obesity, insulin resistance and metabolic syndrome.

## 8. Conclusion

Although it has been known for some time that the microbiota performs numerous biological functions that affect the metabolic functions of the host, it is only within the last number of years that the importance of this is becoming apparent. Our intestinal microbial community can affect the rate of deposition and utilization of fat, insulin resistance and diabetes and our inflammation state, as well as our general health and wellbeing. Over recent years, large observational studies and animals trials in combination with high throughput technologies have been used to identify and understand the impact of environmental factors, such as diet and genotype, on controlling the microbiota. The importance of the association of the microbiota and its functions with diet cannot be over-stated in the light of the monumental shifts in diets that have occurred in a very short evolutionarily timeframe. These diets are continuing to change, and it is now estimated that in the United States, a sizeable proportion of individuals consume very little vegetables, with less than half of individuals reporting eating vegetables at dinner. Even less of these individuals include vegetables in lunch foods, like sandwiches and wraps [119]. It may be that our consumption of processed foods, widespread use of antibiotics and disinfectants and our modern lifestyle may have forever altered our ancient gut microbiome. We may never be able to identify or restore our microbiomes to their ancestral state, but dietary modulation to manipulate specific gut microbial species or groups of species may offer new therapeutic approaches to conditions that are prevalent in modern society, such as functional gastrointestinal disorders, obesity and maybe even age-related under-nutrition. We predict that this will become an increasingly important area of health research.
